# Preliminary Evidence for a Western Blot Diagnosis of Satoyoshi Syndrome Using SH-SY5Y Neuroblastoma Cell Lysate as the Antigen Source

**DOI:** 10.3390/diagnostics15212797

**Published:** 2025-11-05

**Authors:** José María Sevilla Avendaño, Carlos Garrido, Irene Rodríguez Clemente, Julián Solís-García del Pozo, Ulrich Stephani, Ricardo Martínez, Carlos de Cabo, Valentín Ceña, Javier Solera

**Affiliations:** 1 Neuropsychopharmacology Unit, Research Department, Complejo Hospitalario Universitario de Albacete, 02008 Albacete, Spain; chema.lezuza@gmail.com (J.M.S.A.); cgarridot@sescam.jccm.es (C.G.); 2 Immunological, Molecular, and Genetic Bases of Satoyoshi Syndrome Group, Instituto de Investigación Sanitaria de Castilla-La Mancha (IDISCAM), 45004 Toledo, Spain; jesolis@sescam.jccm.es (J.S.-G.d.P.); josejavier.solera@uclm.es (J.S.); 3 Unidad Asociada Neurodeath, Institute of Molecular Nanoscience, INAMOL, Universidad de Castilla—La Mancha, 02008 Albacete, Spain; irene.rclemente@uclm.es (I.R.C.); valentin.cena@uclm.es (V.C.); 4 Department of Internal Medicine, Complejo Hospitalario Universitario de Albacete, 02008 Albacete, Spain; 5 Klinik für Neonatologie, Kinderpneumologie und Neuropädiatrie (Kinder und Jugendmedizin II), University Hospital of Schleswig Holstein (UKSH), Campus of Kiel, 24118 Kiel, Germany; u.stephani@med.uni-kiel.de; 6 Department of Oral and Maxillofacial Surgery, Hospital Universitario de Canarias, 38320 La Laguna, Spain; rimmtf@gmail.com; 7 Department of Medical Sciences, Faculty of Medicine, Universidad de Castilla—La Mancha, 02008 Albacete, Spain

**Keywords:** Satoyoshi syndrome, SH-SY5Y, Western blot, autoimmune, autoantibodies, diagnosis, rare diseases, biomarkers

## Abstract

**Background/Objectives**: Satoyoshi syndrome is a rare, autoimmune disorder currently diagnosed based on clinical criteria: painful muscle spasms, diarrhea, and alopecia. Two previous reports showed a specific immunoreactive band in three Satoyoshi syndrome patients using Western blot analysis, with brain homogenate as the antigen source. These findings could be the basis for a future diagnostic test. The aim of our study was to evaluate the efficacy of using SH-SY5Y cell lysate instead of brain homogenate for a potential laboratory test for Satoyoshi syndrome using the Western blot technique. **Methods**: Western blot analyses were conducted using brain homogenate, SH-SY5Y cell lysates, and differentiated SH-SY5Y cell lysates. Serum samples were obtained from three Satoyoshi syndrome patients, alongside control samples from thirty blood donors and six patients with other neurological conditions. **Results**: Sera from patients with Satoyoshi syndrome displayed a three-band pattern in the 70–100 kDa range. This pattern was reproducible across all tested antigen sources (brain homogenate, SH-SY5Y lysate, and differentiated SH-SY5Y lysate) but was not observed for the sera from the control groups. The bands were more visible when using either type of SH-SY5Y lysate compared to brain homogenate. No differences were found between the SH-SY5Y lysate and the differentiated SH-SY5Y lysate. **Conclusions**: Sera from our Satoyoshi syndrome patients showed a specific band pattern that could be used for a future evaluation of Satoyoshi syndrome using Western blot. The use of SH-SY5Y cell lysate vs. brain homogenate as an antigen source may improve visualization and reproducibility of the immunobands and be less costly.

## 1. Introduction

Satoyoshi syndrome (OMIM: 600705) [1,2] is a rare, multisystemic disorder characterized by a spectrum of clinical features, including painful intermittent muscle spasms, diarrhea, alopecia, skeletal abnormalities, growth retardation, and endocrinopathies [3,4,5,6]. A delay in diagnosis and the subsequent lack of appropriate treatment can lead to poor outcomes, including severe complications and potentially fatal progression [7]. It is believed that Satoyoshi syndrome is an autoimmune disease. The association betweenSatoyoshi syndrome andother autoimmune diseases (thyroiditis, myasthenia, systemic lupus, atopic dermatitis, and idiopathic thrombocytopenic purpura), the presence of autoantibodies (ANAs, anti-acetylcholine receptor (AChR), anti-glutamic acid decarboxylase, among others), and the improvement insymptoms with immune response modifiers are considered evidence supporting an autoimmune origin [3]. The abovementioned serum autoantibodies are not specific to Satoyoshi syndrome. However, an antibody against brain homogenate, which was not found in healthy controls, has been reported in three previous cases using Western blot. Endo et al., 2001 [8], described a 13-year-old female patient with Satoyoshi syndrome who carried the anti-AChR autoantibody, without presenting symptoms of myasthenia gravis. Analysis of this patient’s serum using Western blot on brain homogenate revealed an 85 kDa immunoreactive band, although the specific antigen remained unidentified [8]. Similarly, Matsuura et al., 2007, reported two female patients (aged 17 and 36) with classic symptoms of Satoyoshi syndrome, whose sera showed a 90 kDa band when tested with brain, stomach, and duodenum homogenates using Western blot [9].

The aim of this study was to investigate whether the sera from three otherSatoyoshi patients from Europe also carry specific autoantibodies as assessed by Western blot, using either brain homogenate or SH-SY5Y cell lysate as the antigen source. SH-SY5Y is a well-known human neuroblastoma cell line derived from a bone marrow biopsy of a patient with neuroblastoma. It is widely used in neuroscience research as an in vitro model to study neuronal differentiation, function, and processes related to neurodegenerative diseases, like Parkinson’s disease [10,11,12,13]. This cell line was chosen for this study as an alternative to human brain homogenate because it is well described in the literature, easy to obtain commercially, and, therefore, facilitates the standardization of the procedure.

## 2. Participants and Methods

In a first experiment, we assessed the autoimmune immunoreactivity of the serum from three Satoyoshi patients on brain homogenate and two types of SH-SY5Y cell lysates, namely, standard and after inducing neuronal differentiation, to determine which source of antigen would be more suitable. SH-SY5Y (Cellosaurus database accession number: CVCL_0019); https://www.cellosaurus.org/CVCL_0019 (accessed on 15 September 2025) is a neuroblastoma cell line commonly used as an in vitro model for neurological disease [10,11,12,13]. These cells are susceptible to being differentiated into mature neurons [13]. SH-SY5Y cells were purchased from the American Type Culture Collection, https://www.atcc.org/. In a second experiment, we aimed to compare the immunobands obtained from the serum samples of Satoyoshi patients using Western blot with the immunobands from the sera of healthy blood donors and patients with other neurological diseases to investigate the possibility of a specific band pattern for Satoyoshi patients.

### 2.1. Patient Samples

Serum samples from three patients with previously reported Satoyoshi syndrome were analyzed in this study [14,15,16]. A brief summary of the three cases follows.

#### 2.1.1. Case 1 [14]

The first case was a 28-year-oldwoman from Albacete (Spain), who developed alopecia at 10 years of age. Two years later, she began experiencing progressive, painful muscle spasms, followed by diarrhea with carbohydrate malabsorption, iron-deficiency anemia, weight loss, skeletal abnormalities, and amenorrhea. A definitive Satoyoshi syndrome diagnosis was not reached until the patient was 18 years old. Treatment with prednisone (30 mg/day) and methotrexate (7.5 mg/week) was initiated, with dramatic improvement of muscle spasms, resolution of diarrhea, weight gain of 20 kg, and recovery of menstruation.

#### 2.1.2. Case 2 [15]

The second case was a 17-year-old girl from the Canary Islands (Spain), with a history of asthma and allergic rhinitis, presenting with alopecia universalis at 5 years of age. At 11 years of age, she exhibited painful, intermittent muscle spasms, significant growth retardation, diarrhea, and abdominal pain. Her self-limiting muscle spasms, localized to the acral areas of her limbs, increased with physical activity and typically lasted several minutes. The patient was diagnosed with Satoyoshi syndrome, and treatment with carbamazepine and otilonium bromide led to the disappearance of her muscle spasms and diarrhea.

#### 2.1.3. Case 3 [16]

The third case was a 38-year-old woman from Kiel (Germany). At 13 years of age, she began presenting muscle spasms, with jaw cramps being the most uncomfortable. She was treated with carbamazepine, prednisolone, and sexsteroids. After introducing low doses of methotrexate to the therapy, the patient recovered from muscle spasms, alopecia, and diarrhea. At 18 years of age, methotrexate and corticosteroid medication were tapered off, without relapse. Initiation of sexsteroid treatment resulted in pubertal development and regular menstrual cycles. She has thus far experienced three pregnancies, all terminating in spontaneous abortions.

### 2.2. Control Groups

Two control groups were included as follows: (a) one consisting of 30 blood donors provided by the Albacete General University Hospital Blood Bank, and (b) a second group of patients with other neurological pathologies. The patients included in this second category were a 58-year-old female with radiculopathy, a 41-year-old male with acute disseminated encephalomyelitis, a 55-year-old female with secondary progressive multiple sclerosis, a 73-year-old male with Parkinson’s disease, a 34-year-old female with encephalitis caused by human herpes virus type 6, and a 53-year-old male with demyelinating polyneuropathy. Samples from these patients were provided by the Albacete General University Hospital Biobank.

The present study was compliant with the Declaration of Helsinki, and ethical approval was obtained prior to data and sample collection by the local Medicine Research Ethics Committee of the Albacete General University Hospital (Acta 04/2016 and Acta 10/2021). All participants provided written informed consent for this study.

### 2.3. Laboratory Methods

For the identification of autoantibodies in patient serum, we used whole brain homogenate (Biochain, Newark, CA, USA), lysates from SH-SY5Y (ATCC, Manassas, VA, USA) cells, and differentiated SH-SY5Y cells. The brain homogenate was reconstituted according to the manufacturer’s instructions.

#### 2.3.1. Preparation of SH-SY5Y Cell Lysates

SH-SY5Y cells were cultured in Dulbecco’s Modified Eagle Medium/Ham’s Nutrient Mixture F-12 (DMEM F-12) supplemented with 2 mM L-glutamine (Gibco, Grand Island, NY, USA), 100 U/mL penicillin and 100 U/mL streptomycin (penicillin–streptomycin; Gibco, Grand Island, NY, USA), and 10% (*v*/*v*) heat-inactivated fetal bovine serum (FBS; Gibco, Gaithersburg, MD, USA). Cultures were grown in a humidified incubator at 37 °C under a 5% CO_2_ atmosphere. To induce differentiation, the SH-SY5Y cells were treated with 0.1% retinoic acid (Sigma-Aldrich, Saint Louis, MO, USA) for 5 days, with the medium changed every 2 days. The culture medium of the SH-SY5Y cells was removed, and the cells were washed with ice-cold phosphate-buffered saline (PBS, 0.1 M phosphate, 0.15 M sodium chloride, pH 7.2) and lysed for 5 min in 300 μL of ice-cold lysis buffer (Pierce IP, Thermo Fisher Scientific, Rockford, IL, USA). The cell lysates were centrifuged at 13,000× *g* for 10 min. The supernatants were kept for Western blot analysis, and the pellets discarded.

#### 2.3.2. Western Blot

Protein concentrations were determined spectrophotometrically using the Micro BCA Protein Reagent Kit (Thermo Fisher Scientific, Rockford, IL, USA). A total of 25 µg of protein from brain homogenate, SH-SY5Y lysate, and differentiated SH-SY5Y lysate were separated by electrophoresis on 8% SDS-PAGE gels for 30 min at 60V and 1.5–2 h at 100V. Following electrophoresis, the proteins were transferred to nitrocellulose membranes (Immobilon; Millipore Corporation, Billerica, MA, USA) previously activated with transfer buffer [3 g of Base Trizma (Sigma-Aldrich, Saint Louis, MO, USA), 14.4 g of glycine (Sigma-Aldrich, Saint Louis, MO, USA), 200 mL of methanol, and 800 mL of milliQ water], in a Trans-Blot^®^ SD Semi-Dry Transfer Cell (Bio-Rad, Hercules, CA, USA) at a constant amperage of 20 mA for 1.5 h. Nonspecific protein binding was blocked using a blocking buffer, 10% *w*/*v* skimmed milk, and 0.1% Tween 20 (Sigma-Aldrich, Saint Louis, MO, USA) in PBS for 1 h at room temperature (RT). The membranes were incubated overnight at 4 °C with patient serum samples or control serum samples, diluted 1:50 in a blocking buffer. After washing with PBS containing 0.1% *v*/*v* Tween 20, 5 times for 5 min, the membranes were incubated with an anti-human IgG secondary antibody (anti-human IgG (Fc specific)−peroxidase antibody produced in goat; Sigma-Aldrich, Saint Louis, MO, USA), diluted 1:1000 in blocking buffer at RT and washed again with PBS containing 0.1% *v*/*v* Tween 20, 5 times for 5 min. Antibodies were detected using an enhanced chemiluminescence detection kit (GE Healthcare, Little Chalfont, Buckinghamshire, UK). The images were acquired using atransilluminator (Luminescent Image Analyzer LAS-4000 mini, Fujifilm, Tokyo, Japan) with the image capture software LAS-4000 Image Reader (Fujifilm, Tokyo, Japan).

## 3. Results

**Figure 1:**
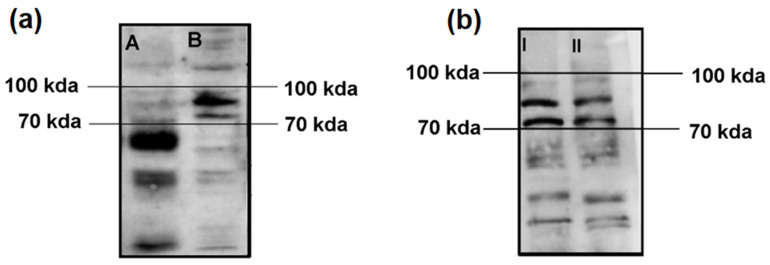
Comparisons of Western blot immunoreactivity results using brain homogenate vs. SH-SY5Y lysate and SH-SY5Y lysate vs. differentiated SH-SY5Y lysate. (**a**) After incubation with serum from the case 1 Satoyoshi patient using brain homogenate (A) and SH-SY5Y cell lysate (B), a common band pattern between 70–100 kDa was found. (**b**) Comparison between the SH-SY5Y cell lysate (I) and the SH-SY5Y differentiated cell lysate (II) revealed the same band pattern between 70 and 100 kDa. A longer running time (2 h vs. 1.5 h) was employed for this blot to allow for a greater separation of the bands.

In our first experiment, we assessed the autoimmune immunoreactivity of the serum from the Satoyoshi patients in brain homogenate and SH-SY5Y cell lysates. We observed a pattern of three bands between 70 and 100 kDa in both the brain homogenate and SH-SY5Y lysate samples. A higher intensity and definition of the bands was observed for the SH-SY5Y lysate (Figure 1a). An additional Western blot was performed to compare the SH-SY5Y lysate and the differentiated SH-SY5Y lysate. The same band pattern was found for both lysates, with no differences in band intensity or definition (Figure 1b).

**Figure 2:**
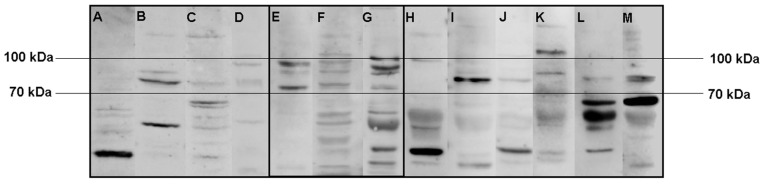
Comparisons of Western blot immunoreactivity band patterns between patients with Satoyoshi syndrome and control groups using SH-SY5Y lysate. After incubation with serum from blood donors (**A**–**D**), serum from Satoyoshi patients ((**E**) case 1, (**F**) case 2, (**G**) case 3), and serum from patients with other neurological conditions (**H**–**M**) using SH-SY5Y cell lysate, a specific band pattern between 70 and 100 kDa was found for the Satoyoshi patients. This pattern was not observed for either control group.

In the second experiment using only SH-SY5Y lysate samples, we observed a common band pattern between 70 kDa and 100 kDa for the serum samples of the three Satoyoshi syndrome patients (Figure 2, E = case 1, F = case 2, G = case 3). This band pattern was absent in the blood donors (Figure 2A–D) and the patients with other neurological conditions (Figure 2H–M), suggesting that this band pattern is specific to Satoyoshi syndrome. No other specific band pattern was found in the blood donors or the other neurological conditions group.

## 4. Discussion

We were able to identify a characteristic band pattern in our three patients with Satoyoshi syndrome, which was more visible using the SH-SY5Y cell lysate than the brain homogenate. No differences in band intensity or definition were found between the SH-SY5Y and differentiated SH-SY5Y cell lysates. This immunoreactive band pattern was absent in the serum of blood donors or patients with other neurological conditions. No specific band pattern was expected in either the donor group or in the patients with other neurological diseases. Healthy individuals may present variations in their serum circulating antibodies, and the neurological patients presented different diseases from each other; therefore, a similar pattern among them could not be presumed. The band pattern found in our study for our three Satoyoshi patients, between 70 and 100 kDa, is within the range of the bands described by Endo et al. (85 kDa) and Matsuura et al. (90 kDa), who employed a similar Western blot technique [8,9]. SH-SY5Y cells express a large number of proteins with molecular weights between 70 and 100 kDa, including those related to neurotransmission, neuronal growth, cell proliferation, cell adhesion, the cytoskeleton, and stress response, among others [10,11,12,13]. Further analysis using additional techniques will be required to confirm the results and determine which specific proteins are targeted by the autoantibodies produced by patients with Satoyoshi syndrome.

The serum samples in this study were obtained at least 5 years after the onset of the disease, when the patients had already received treatment and no longer showed symptoms. The persistence of these characteristic autoantibodies after treatment may allow a retrospective diagnosis. The symptomatology of Satoyoshi syndrome takes years to fully develop, and the appearance of the different symptoms is heterogeneous across patients. Up to now, diagnosis has been based on a typical clinical pattern. A future Western blot test could provide an early diagnosis when patients only present with alopecia or muscle spasms, for example. Satoyoshi syndrome is a very rare disease; therefore, patients are very few and dispersed across the world, making it very difficult to obtain samples. However, samples from more patients are needed to prove the validity of the test. We hope that this preliminary evidence will encourage other professionals to contribute new samples and collaborate with our research. Additionally, efforts should be made to identify the autoantibodies responsible for the immunoreactivity we found, as well as the target protein or proteins.

## 5. Conclusions

In summary, we present a first preliminary approach suggesting that the Western blot technique could be used as a laboratory test to assist in Satoyoshi syndrome diagnosis. SH-SY5Y lysate is a suitable antigen alternative to human brain homogenate when using this technique. The advantages of SH-SY5Y lysate include improved band visualization, better reproducibility, and lower cost. Additional studies involving more Satoyoshi syndrome patients are needed to validate our results.

## Data Availability

The raw data supporting the conclusions of this article will be made available by the authors, without undue reservation.

## Abbreviations

The following abbreviations are used in this manuscript:

SH-SY5Y	Human-derived neuroblastoma cell line used extensively in scientific research
ANAs	Antinuclear antibodies
AChR	Anti-acetylcholine receptor
SDS-PAGE	Sodium dodecyl sulfate–polyacrylamide gel electrophoresis
